# Sex differences in song syntax and syllable diversity in testosterone-induced songs of adult male and female canaries

**DOI:** 10.1186/s13293-023-00533-8

**Published:** 2023-08-01

**Authors:** Ednei B. dos Santos, Gregory F. Ball, David M. Logue, Charlotte A. Cornil, Jacques Balthazart

**Affiliations:** 1 Laboratory of Behavioral Neuroendocrinology, GIGA Neurosciences, University of Liege, 15 Avenue Hippocrate (Bat. B36), Sart Tilman, 4000 Liège 1, Belgium; 2 Program in Neuroscience and Cognitive Science; Department of Psychology, University of Maryland, College Park, MD USA; 3 Department of Psychology, University of Lethbridge, Lethbridge, AB Canada

**Keywords:** Song diversity, Syllable sequences, Sex differences, Testosterone, Network analysis

## Abstract

**Background:**

Behavioral sex differences are widespread in the animal world. These differences can be qualitative (i.e., behavior present in one sex but not the other, a true sex dimorphism) or quantitative (behavior is present at a higher rate or quality in one sex compared to the other). Singing in oscine songbirds is associated with both types of differences. In canaries, female rarely sing spontaneously but they can be induced to do so by treatments with steroids. Song in these females is, however, not fully masculinized and exhibits relatively subtle differences in quality as compared with male song. We analyzed here sex differences in syllable content and syllable use between singing male and female canaries.

**Methods:**

Songs were recorded from three groups of castrated male and three groups of photoregressed female canaries that had received Silastic™ implants filled with testosterone (T), with T plus estradiol (E2), or left empty (control). After 6 weeks of hormone treatment, 30 songs were recorded from each of the 47 subjects. Songs were segmented and each syllable was annotated. Various metrics of syllable diversity were extracted and network analysis was employed to characterize syllable sequences.

**Results:**

Male and female songs were characterized by marked sex differences related to syllable use. Compared to females, males had a larger syllable-type repertoire and their songs contained more syllable types. Network analysis of syllable sequences showed that males follow more fixed patterns of syllable transitions than females. Both sexes, however, produced song of the same duration containing the same number of syllables produced at similar rates (numbers per second).

**Conclusions:**

Under the influence of T, canaries of both sexes are able to produce generally similar vocalizations that nevertheless differ in specific ways. The development of song during ontogeny appears to be a very sophisticated process that is presumably based on genetic and endocrine mechanisms but also on specific learning processes. These data highlight the importance of detailed behavioral analyses to identify the many dimensions of a behavior that can differ between males and females.

**Supplementary Information:**

The online version contains supplementary material available at 10.1186/s13293-023-00533-8.

## Plain Language Summary

Male canaries normally sing complex songs at high rate while females only rarely sing very simple songs. Testosterone induces active singing in both male and female canaries, but female song is still not fully masculinized by these treatments even if song duration does not differ between the sexes. We analyzed the syllable repertoire and the sequence of use for different syllables in canaries of both sexes treated with testosterone or testosterone supplemented with estradiol. Compared to females, males had a larger syllable-type repertoire and their songs contained more syllable types. Syllable transitions were also more fixed in males. Sex differences in adult singing of canaries are thus a complex mixture of differences that result from the different endocrine condition of males and females (and are thus partially reversed by administration of exogenous testosterone) and of more stable differences that presumably develop during the ontogenetic process under the influence of endocrine and genetic differences and of differential learning processes. Canary song thus represents an outstanding model system to analyze the interaction between nature and nurture in the acquisition of a sophisticated learned behavior as well as the mechanisms controlling sex differences in vocal learning and production.

**Supplementary Information:**

The online version contains supplementary material available at 10.1186/s13293-023-00533-8.

## Highlights

- Testosterone activates active singing in male and female canaries.
- Male songs contain a larger syllable repertoire compared to females.
- Syllable sequences in male songs are produced with more fixed transitions and less flexibility.
- Syllable repertoire does not seem to correlated with the volume of vocal control nuclei.

**Supplementary Information:**

The online version contains supplementary material available at 10.1186/s13293-023-00533-8.

## Introduction

Birdsong and the supporting network of brain nuclei, the vocal control system, have emerged as a widely used model system to analyse biological mechanisms underlying important phenomenon including vocal learning, brain plasticity, brain lateralization, steroid action on brain and behavior, and also sex differences in brain and behavior (reviewed in [1–7]). In many songbird species including canaries, syllables are building blocks, and can come in different types. Song diversity arises from the combination and order that different syllable types are used in song construction. These syntax-like combinatorial properties have often been compared to human language (reviewed in [2, 3]).

Complex singing patterns are hypothesized to function as signals for male quality assessment by rivals and/or potential mates [8, 9]. Females that choose males that sing more diverse (or complex) songs may mate with higher quality males and obtain greater direct benefits from them, such as better parental care and a larger territory with more resources, or greater indirect benefits in the form of superior genes that improve offspring in various ways including enhanced immunocompetence [10, 11]. In turn, males that sing more diverse songs might acquire extra-pair fertilizations, multiple mates, or mates of superior quality and have increased reproductive success as a result. In canaries (*Serinus canaria*) specifically, Kroodsma showed in a classic study [12] that females build nests faster and lay more eggs when exposed to playbacks of male songs with high syllable diversity compared to songs with a smaller repertoire.

It was previously shown that male songs containing syllables with a more complex structure (two-note structure, high repetition rate, rapid frequency modulation and short intervals between syllables) are preferred by females [13]. These syllables called “sexy syllables” induce in females a higher activation of secondary auditory areas as measured by the induction of immediate early gene expression as compared to male songs without these syllables [14]. In addition, songs produced by T-treated female canaries were recently shown to be able to enhance immediate early gene expression in the auditory forebrain of other females, but it is not known if this expression is equivalent to what happens in response to male song [15].

Studies on birdsong are historically male-biased, partly because research has focused on northern temperate zone species, in which males sing more often than females, or on zebra finches, a species in which females do not sing at all [16, 17]. However, recent studies have shown that song in both sexes is probably the ancestral condition in songbirds (suborder Passeri) and that female song is much more common than previously thought [18, 19].

Female song has, therefore, been largely understudied and in particular, studies reporting quantitative information about sex differences in song and syllable-type usage are rare, presumably due, at least in part, to the fact that cataloguing and annotation of syllable-type repertoires are much more time-consuming than scoring time (rate, duration) and frequency domain of song variables. In the present study, we partly address this gap in the literature by providing a thorough analysis of song and syllable diversity in the testosterone-induced songs of male and female canaries.

Both male and female canaries sing but spontaneous female song is both more plastic and much less common than male song. A recent study characterizing spontaneous female song in canaries estimated that less than 7% of females raised in aviary conditions sing [20]. However, female canaries can be induced to sing by treatments with exogenous steroid hormones. After 2 to 3 weeks of testosterone (T) treatment, females start singing at high rates [21, 22]. As a consequence, T-treated female canaries are commonly used in experiments assessing the effects of sex steroids on singing behavior and brain anatomy [23, 24]. This might, however, not reflect precisely what happens in males at the beginning of the breeding season when endogenous T concentrations start increasing. Indeed, we previously reported in a study based on the same birds and song recordings [22] that several measures of singing behavior and song structure are activated more efficiently both by T and T plus estradiol (E2) treatments in male than in female canaries. These treatments with steroids initiated in adulthood also increase the volume of forebrain song control nuclei (HVC, RA, and Area X) in both sexes, but these nuclei remain significantly smaller in females than in males irrespective of treatment [22]. Additional analyses of songs from the same birds also suggested that sex differences in trill production are related to sex differences in syrinx mass. Males produce songs with more trills and have heavier syrinxes and these differences cannot be reversed by sex steroids in adulthood [25].

During the analysis of these vocalizations, we also noticed that female songs induced by the steroid treatments seemed to contain a much smaller number of different syllables. To follow up on this observation, we examined here potential sex differences in syllable diversity and use in male and female Fife fancy canaries that had been treated with sex steroid hormones. Given that control males and females in this experiment did not sing at all or only sang very rarely, the present analyses only concern the steroid-treated birds. We included, however, the two different treatments, with T or with T supplemented with E2, in the analyses to further test whether addition of E2 could improve female songs. In addition to the song and syllable diversity analysis, we employed a set of network analysis tools to visualize and quantify variability in syllable-type sequences [26–29].

## Materials and methods

### Study subjects

The present analyses are based on recordings that were collected in a published experiment [22]. These analyses were conducted, because our previous work suggested the existence of sex differences in the syllable repertoire of males and females treated with T in adulthood. They were, therefore, not planned initially (not hypothesis-driven) but represent a post-hoc exploratory study. Experimental procedures were previously described in detail in a published paper [22] and are just briefly summarized here.

One-year-old Fife Fancy canaries (23 females and 24 males) were acquired from a commercial breeder in Antwerp, Belgium. We housed birds indoors in wire cages (49 × 95 × 51 cm) at the University of Liege, Belgium. Males and females were kept in separate cages in groups of six individuals on an 8 L:16 D (light/dark) cycle that was maintained throughout the experiment. They received seed mix and water ad libitum, as well as baths and grit daily, and eggfood (blended hard-boiled chicken eggs and bread) twice a week. The sex of birds was determined via molecular sexing at the Behavioral Ecology and Ecophysiology lab animal facility of the University of Antwerp, Belgium [30].

### Surgical procedures

Six weeks after arriving at our animal facilities, males were castrated under general anesthesia induced by a mix of air gas and 3% isoflurane for induction and 2.5% for maintenance. Testes were regressed at the time of castration, which indicates that males were in non-breeding condition. Testes were removed through a small unilateral incision in the left flank, between the last two ribs. After suturing the incision, we allowed males to recover under a warm light before returning them to their cages. Females were not ovariectomized but were laparotomized to confirm that ovaries were photoregressed. The high level of vascularization of the ovary in female canaries makes it difficult to perform ovariectomies without inducing a high rate of mortality. Previous studies have successfully used intact females that had photoregressed ovaries, so our approach facilitates comparison with previous work [21, 22, 30, 31]. This ovarian regression spontaneously occurs when birds are exposed to short daily photoperiods. As a result, subjects have low circulating concentrations of sex steroids and become sexually non-responsive. Females were similarly returned to their home cages after they had recovered from surgery under a warm lamp.

### Hormone implants

Three weeks after surgeries, subjects received subcutaneous implants made with Silastic ™ tubing (Dow corning, Midland, MI, USA; Degania Silicone; internal diameter 0.76 mm, external diameter 1.65 mm, length 10 mm) filled with either crystalline steroids or left empty as a control. Implants were pre-incubated in 0.9% saline at 37 °C overnight and inserted subcutaneously through a small hole in the back of the birds. Subjects were kept under isoflurane anesthesia during the surgical procedure and the hole was sutured with a thread (5-0 coated Vicryl™). Birds (*n* = 8 of each sex per experimental group) were implanted with Silastic™ implants filled with either T or T+E2, or left empty (control). The T+E2 group was included, because previous studies reported that the induction of aromatase activity by T is lower in the female than in the male brain in all mammalian and avian species that have been studied (see [22] for references). This sex difference in aromatase activity might explain the limited behavioral response of females to exogenous T [21, 22], since activation of several features of song is mediated by estrogens derived from T aromatization [32–35]. Therefore, we added exogenous E2 in an effort to compensate for this possible sex difference. Although our previously published study [22] identified some effects of E2 on general features of song, the comparison of the treatments with T and T+E2 did not reveal any effect of E2 on the dependent variable considered in the present analyses. We document this lack of treatment effect in “Results” section.

### Song recordings and annotations

Six weeks after the onset of steroid treatment each bird was recorded for 3 h starting immediately after the lights went on (09:00 h). Individual birds were placed on the previous day inside custom-built sound-attenuated boxes. Songs were recorded using custom-made microphones (Projects Unlimited/Audio Products Division) and an Allen & Heath ICE-16 multichannel recorder. The sound files were acquired and saved as wav files by Raven v1.4 software (Bioacoustics Research Program 2011; Raven Pro: Interactive Sound Analysis Software, Version 1.4, Ithaca, NY: The Cornell Lab of Ornithology) at a sampling frequency of 44,100 Hz which translates to a frequency range of 0–22,050 Hz.

We analysed the first 30 songs produced by each individual. Vocalizations were defined as songs if they were at least 1 s long and were preceded and followed by at least 0.4 s of silence as done in our previous work [22]. We defined a syllable as a single or grouped combination of sounds that are consistently produced together as a common unit [36, 37]. We defined syllable types based on their appearance on a sound spectrogram to develop a catalog of syllables for each individual. Songs were then segmented and their syllable content annotated using PRAAT, version 6.2.23 (see https://www.fon.hum.uva.nl/praat/).

### Diversity metrics

We measured eight variables that are conventionally used to capture song diversity (or complexity) at multiple levels [38, 39]: *song repertoire size* (number of different song types), *syllable-type repertoire size* (number of different syllable types), *number of syllables per song*, *number of syllable types per song*, *number of syllable types per second*, *number of syllables per second*, *syllable versatility index* (SVI; number of syllable types/number of syllables) and *Levenshtein distance*. Levenshtein distance measures variation in syllable-type sequences between successive songs. It does this by quantifying the minimum number of edits required to convert a string of characters, such as a sequence of syllables, into another using single-place deletions, insertions or replacements of individual characters [40]. Some of these dependent variables are obviously correlated, especially those reflecting the number of different syllable types. We elected to provide data on each of them to fully document the fact that the sex difference is genuine and does not relate only to a specific way of measuring syllables diversity. These correlations were actually calculated and submitted to a principal component analysis to assess their relationships with vocal control nuclei (see end of “Results” section).

### Network analysis of syllable-type sequences

Based on graph theory, network analysis has been applied to a wide range of scientific disciplines, from molecular networks to social organization. In animal communication, network analysis tools have been used as a complement to more traditional measures of song diversity to assess properties of song organization and diversity that are not revealed by other approaches [29], including whether patterns of syllable transitions are more linear and fixed or more variable.

Thus, in addition to the diversity metrics listed above, we analysed the sequences of syllable types using a network-based approach [26, 27, 29]. Syllable-type sequence networks were created for each individual from a string of successive syllable types over a sample of 30 songs (the same used for the previous set of analyses). The breaks between songs were ignored to avoid creating small, disconnected networks [29]. Networks comprised of three or fewer syllable types were removed from further analysis to avoid bias resulting from the limited number of nodes [29].

Syllable-type networks were created in R (R Core Team 2022) using the igraph package [41]. Calculations were based on undirected networks. We assigned syllable types as the nodes and the first-order transitions between syllable types as the edges of the network. To quantify transition relationships between syllable types, we used two network variables: the *average minimum path length (path length in short)* and the *network density*. These metrics allowed us to characterize song structure by the organization of its basic components and measure flexibility of syllable-type transitions [29].

*Average path length* is the average of the minimum number of edges (transitions) required to link any pairs of nodes (syllable types) in a network while ignoring dead ends. We decided here to keep the repeats in the results, since we were mainly interested in the global structural differences that characterize syllable transitions in male and female songs. In addition, repeated paths are common occurrences in canary song. By including repeats, we can capture the inherent structure of canary singing style in a more accurate way. Higher values of average path length indicate that more transitions are required to connect any given pair of syllable types. This suggests that syllable sequences are produced in a more constrained or fixed fashion; different syllables are produced in relatively fixed sequences and there is less connectivity between syllable types. Conversely, shorter average path lengths indicate less regularity in syllable-type sequences. In other words there is more flexibility in the transitions between different song types.

Another measure of networks reported here is the *Network density* which is computed as the ratio of the number of edges observed in the network divided by the number of all possible syllable transitions in the network given its size. Here, higher values indicate that more transitions do occur, so that syllable sequences are more flexible, while lower values indicate more limited transitions and thus more stereotypy (see [29] for more detail).

### Statistical analysis

Unless otherwise mentioned, data were analyzed by one- or two-way analyses of variance (ANOVA), or by two-way mixed model analysis, if a few data points were missing. The three experimental groups and two sexes were considered as independent factors. Statistical analyses were performed using GraphPad Prism version 8.2.1 for Mac (GraphPad Software, San Diego, California USA). Effect sizes were calculated as partial eta square for each independent variable and their interaction by calculating the ratios of relevant sums of squares. Correlations between dependent variables were calculated with the Pearson’s product-moment coefficient and subjected to a principal component analysis to reduce the dimensions of the dependent measures. The three first factors extracted by the analysis were then related to the volume of vocal control nuclei as measured in our previous study on the same birds [22]. We used an alpha level of 0.05 for all statistical tests. All data are represented here by their mean ± SEM.

## Results

The total sample comprised 47 individuals (24 males and 23 females). Songs from control birds were excluded from the analysis, because they were very rare and were mostly characteristic of the plastic phase of development when syllables that are produced are not yet stereotyped [42, 43]. Hence, it was not possible to clearly delineate their syllable content. Three females and one male also sang at very low rates even after T treatment. It was therefore impossible to obtain a sample of 30 songs in the recordings of these birds collected after 6 weeks of treatment. These birds were also excluded from the analysis. The final sample comprised a total of 840 songs (57,872 syllables) from 15 males and 13 females from the T and T+E2 groups (see Additional file 1: Table S1 for details). This study thus essentially analyzes the sex differences in repertoire that are revealed by the steroid treatment.

### Male songs show higher diversity

**Fig. 1:**
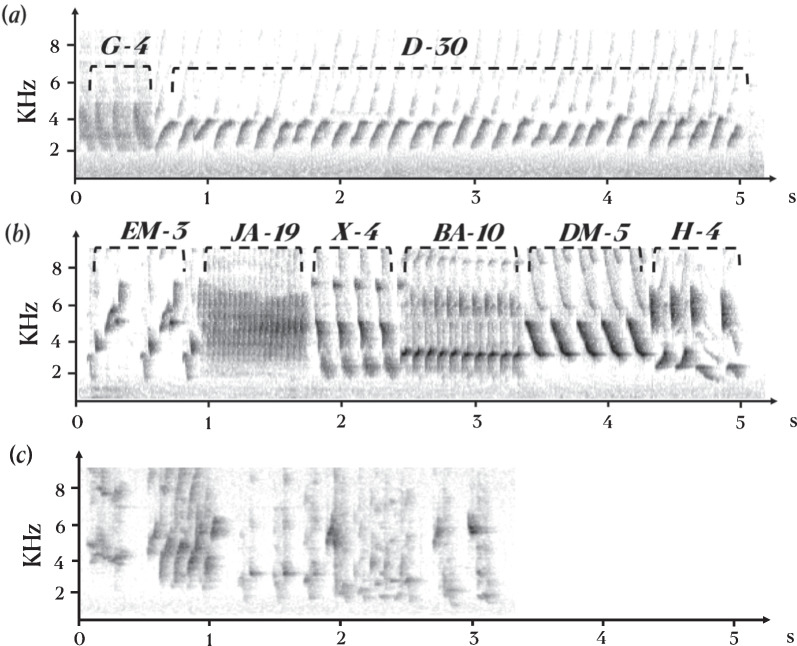
Spectrograms of canary songs after 6 weeks of treatment with steroids. **a** Female, **b** male, **c** typical plastic song from control male with unclear syllable shapes that could not be classified. The different syllable types were catalogued and labeled based on clear and consistent differences in overall temporal–spectral shape that were repeatable across songs both within and between birds. The labels of the different syllable types are indicated above the sonograms

Spectrograms indicated the presence of stereotyped repeatable syllables in the songs of males and females treated with T or T and E2 but not in control birds (Fig. 1).

No qualitative difference could be detected between the two steroid-treated groups (T vs. T+E2) within each sex. In particular songs in the two treatment groups were crystallized and contained easily recognizable syllables. Very long songs were also produced independent of the treatment; singing power and distribution of energy across frequencies were also similar (see [22] for a quantitative analysis of all these features).

**Fig. 2:**
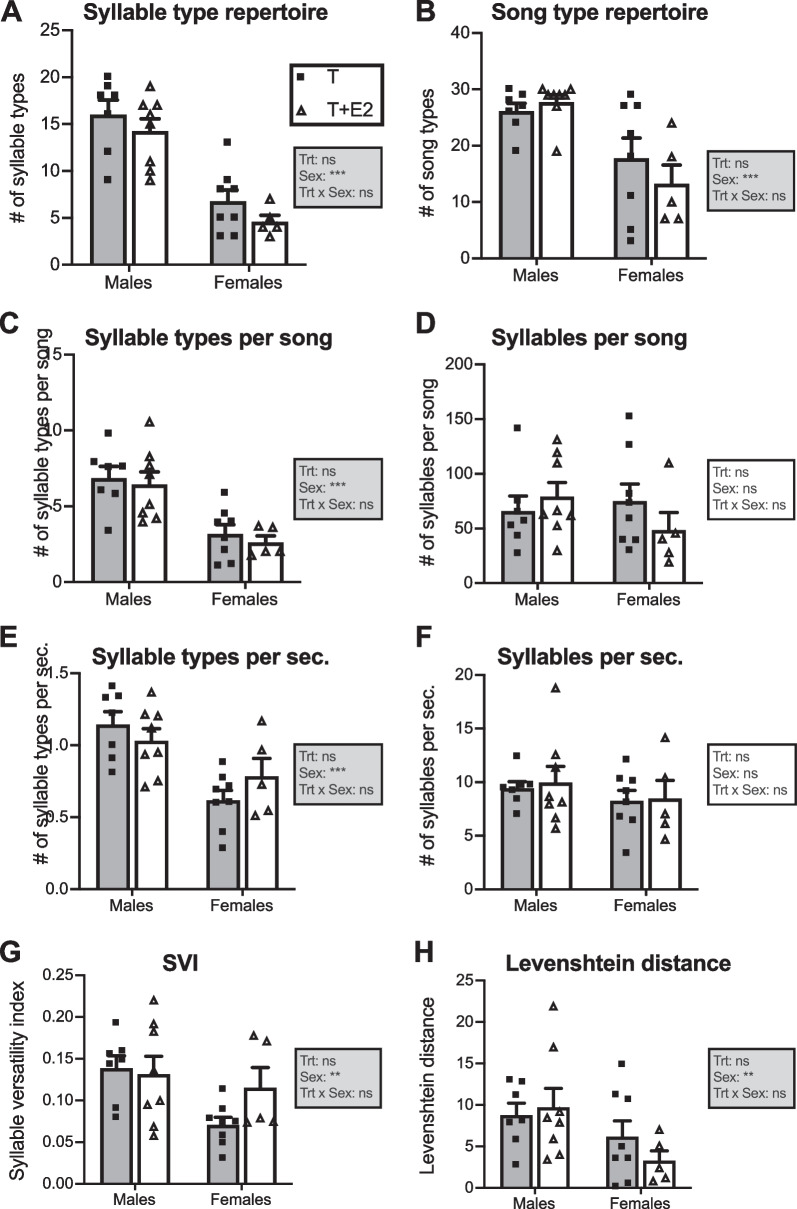
Song and syllable diversity metrics in male and female canaries that were treated with Silastic™ implants filled with testosterone (T; gray bars) or with testosterone plus estradiol (T+E2; white bars). Bar graphs represent the mean ± SEM of results that are the average of the first 30 songs produced by each experimental individual after 6 weeks of steroids treatment. Individual data points are also indicated. Data were analyzed by two-way ANOVA with treatment (Trt) and Sex of the subjects as independent factors and results are summarized in the inset for each panel (***p < 0.001, **p < 0.01, *p < 0.05, ns = not significant). The insets have a gray background when showing a sex difference and a white background when no such difference is present. The other inset in panel A indicates the code for the two experimental groups. See Table 1 for the detail of statistical results

Quantitative analyses of eight variables characterizing these songs by two-way ANOVAs (treatment and sex as independent variables) identified no overall effects of the treatments by steroids (T vs. T+E2) and no significant interaction between the two factors (see Fig. 2 and Table 1). In contrast, these ANOVAs detected significant sex differences for six variables: syllable-type repertoire (Fig. 2A), song-type repertoire (Fig. 2B), numbers of syllable types per song (Fig. 2C), and numbers of syllable types per second (Fig. 2E) were significantly larger in males than in females. This was also the case for the Syllable Versatility Index, SVI (Fig. 2G) and Levenshtein distance (Fig. 2H). However, ANOVAs did not reveal significant sex differences in the number of syllables per song (Fig. 2D) or the number of syllables produced per second (Fig. 2F).

**Table 1:** Results of the two-way ANOVAs comparing multiple song metrics in males and females treated for 6 weeks with T or T+E2

Variable	Trt			Sex			Int		
	*F*_df_	*P*	*η*_p_^2^	*F*_df_	*P*	*η*_p_^2^	*F*_df_	*P*	*η*_p_^2^
Syllable-type repertoire	*F*_1,24_ = 2.51	0.155	0.082	*F*_1,24_ = 50.53	0.0001***	0.678	*F*_1,24_ = 0.226	0.882	0.001
Song-type repertoire	*F*_1,24_ = 0.306	0.585	0.013	*F*_1,24_ = 18.62	0.0002***	0.437	*F*_1,24_ = 1.341	0.258	0.053
Syllable types/song	*F*_1,24_ = 0.452	0.508	0.018	*F*_1,24_ = 26.20	0.0001***	0.522	*F*_1,24_ = 0.008	0.928	< 0.001
Syllables/song	*F*_1,24_ = 0.199	0.659	0.008	*F*_1,24_ = 0.526	0.4754	0.021	*F*_1,24_ = 1.782	0.194	0.069
Syllable types/s	*F*_1,24_ = 0.091	0.766	0.004	*F*_1,24_ = 18.66	0.0002***	0.437	*F*_1,24_ = 2.420	0.133	0.092
Syllables/s	*F*_1,24_ = 0.091	0.766	0.004	*F*_1,24_ = 1.127	0.2989	0.045	*F*_1,24_ = 0.021	0.886	0.001
SVI	*F*_1,24_ = 1.085	0.308	0.043	*F*_1,24_ = 5.532	0.0272*	0.187	*F*_1,24_ = 2.076	0.162	0.080
Levenshtein distance	*F*_1,24_ = 0.250	0.622	0.010	*F*_1,24_ = 5.362	0.0294*	0.183	*F*_1,24_ = 0.968	0.335	0.039
Network path	*F*_1,22_ = 0.465	0.502	0.021	*F*_1,22_ = 20.07	0.0002***	0.477	*F*_1,22_ = 0.520	0.478	0.023
Network density	*F*_1,22_ = 0.557	0.463	0.025	*F*_1,22_ = 21.90	0.0001***	0.499	*F*_1,22_ = 0.281	0.602	0.013

The data reports the *F* ratio, degrees of freedom and associated probabilities for the 2 main factors (treatment, i.e., T vs. T+E2 and sex) as well as their interaction. The effect size (partial eta square *η*_p_^2^) is also indicated in each case. Usual interpretations of effect sizes are: 0.01 = small, 0.06 = medium and 0.14 = large effect

* Significant probabilities (*p* < 0.05)

Female thus sang songs of the same overall length as males (as previously reported in [22]) that contained the same number of syllables produced at the same rate (numbers per second), but these female songs contained many repeats of the same syllable types, as clearly visible in Fig. 1.

### Network analysis: male songs show higher linearity in syllable-type transitions

**Fig. 3:**
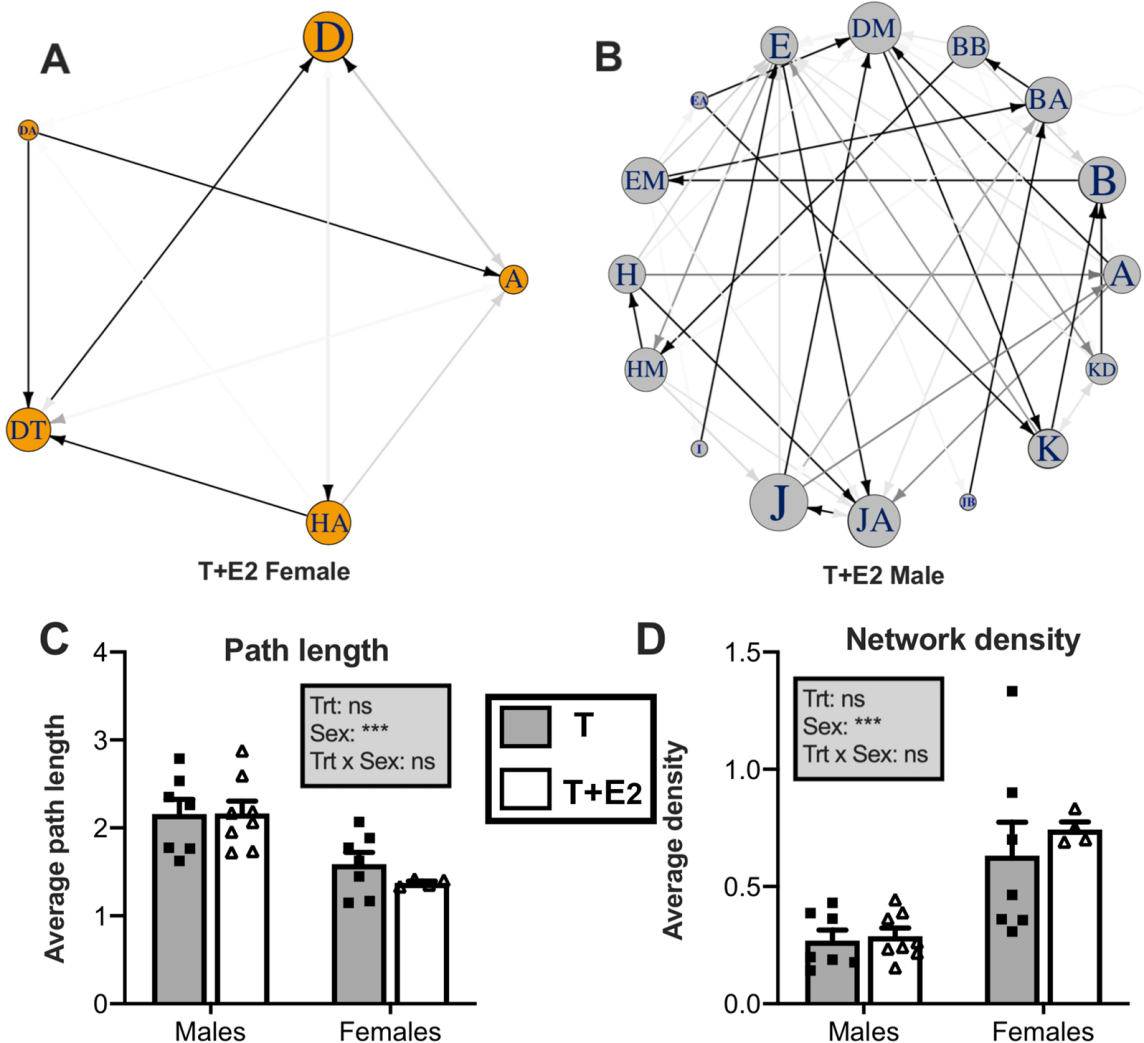
Examples of diagrams illustrating difference in syllable-type use in female (**A**) and male (**B**) canaries treated with sex steroids. Each panel is constructed from a string of successive syllable types over a sample of 30 songs, and the first-order transitions between them. Each circle represents a different syllable type, and differences in the size of the circles capture variation in their relative use. Lines between circles represent syllable-type switching, with the arrow indicating the direction of the transition and the darkness of the line is proportional to the relative frequency of that particular transition. **C**, **D** Illustrate the quantitative sex differences in syllable-type use. Bar graphs represent the mean ± SEM of results. Individual data points are also indicated. Data were analyzed by two-way ANOVA with treatment (Trt) and Sex of the subjects as independent factors and results are summarized in the insert for each panel (***p < 0.001, **p < 0.01, *p < 0.05, ns = not significant)

Representative examples of networks computed for a female and a male of the T+E2 group are presented in Fig. 3A, B. There were significant sex differences for all both network metrics that were used to characterize linearity in syllable-type transitions: overall network path length was higher in males than in females (*F*_1, 24_ = 25.38, *p* < 0.0001, Fig. 3C), while the opposite was seen for network density (*F*_1, 24_ = 40.37, *p* < 0.0001, Fig. 3D). There were no significant treatments or interaction effects (all *p* > 0.33).

### Relationships with volumes of vocal control nuclei

Our previous study of the same birds had also determined the volume of vocal control nuclei, HVC, RA and Area X. We wondered whether the individual differences in repertoire observed here were in any way related to these measures. Because the different measures of repertoire size were, as already mentioned, obviously correlated, we first computed the correlations between these dependent variables after a standardization to a mean of 0 and a standard deviation of 1. The standardized measures were then subjected to a unrotated principal component analysis. The first 3 factors (PC1 to 3) that were extracted explain 90.6% of the total variance (cumulative eigenvalues, respectively 0.514, 0.252 and 0.14 for PC1 to 3) and were therefore used to assess the relationships with vocal control nuclei volumes.

We developed for each sex three linear regression models using PC1, PC2, and PC3 as dependent variables and the vocal control nuclei volumes (HVC, RA and Area X) as predictors, with body mass as a co-variate. Results of these regression analyses are shown in Table 2.

**Table 2:** Results of the multiple linear regression models of the syllable repertoire (first 3 PC of the Principal component analysis) on predictor variables (volume of 3 vocal control nuclei with whole body mass as co-variate), including for each predictor the estimate and associated probability

	Males			Females		
PC1	(*F*_4,7_ = 0.78, *R*^2^ = 0.31, *p* = 0.569)			(*F*_4,4_ = 1.459, *R*^2^ = 0.58, *p* = 0.362)		
Predictors	Estimate (SE)	*t*	*p*	Estimate (SE)	*t*	*p*
HVC volume	− 2.151 (4.903)	− 0.44	0.674	8.637 (6.93)	1.25	0.281
RA volume	6.024 (10.236)	0.59	0.575	16.259 (15.435)	1.05	0.352
X volume	4.131 (3.815)	1.08	0.315	2.679 (5.234)	0.51	0.636
Body mass	0.151 (0.135)	1.11	0.302	0.315 (0.249)	1.26	0.275
Constant	− 4.807 (3.494)	− 1.38	0.211	− 12.156 (5.818)	− 2.09	0.105

PC2	(*F*_4,7_ = 0.08, *R*^2^ = 0.04, *p* = 0.985)			(*F*_4,4_ = 2.13, *R*^2^ = 0.68, *p* = 0.24)		
Predictors	Estimate (SE)	*t*	*p*	Estimate (SE)	*t*	*p*
HVC volume	0.064 (7.588)	0.01	0.993	16.6 (6.401)	2.59	0.06
RA volume	5.472 (15.842)	0.35	0.74	4.154 (14.256)	0.29	0.785
X volume	− 1.051 (5.905)	− 0.18	0.864	− 0.654 (4.835)	− 0.14	0.899
Body mass	− 0.073 (0.21)	− 0.35	0.737	0.072 (0.23)	0.31	0.772
Constant	0.439 (5.408)	0.08	0.938	− 5.047 (5.374)	− 0.94	0.401

PC3	(*F*_4,7_ = 4.63, *R*^2^ = 0.73, *p* = 0.038*)			(*F*_4,4_ = 1.01, *R*^2^ = 0.50, *p* = 0.495)		
Predictors	Estimate (SE)	*t*	*p*	Estimate (SE)	*t*	*p*
HVC volume	2.811 (3.266)	0.86	0.418	− 2.87 (4.837)	− 0.59	0.585
RA volume	− 0.097 (6.819)	− 0.01	0.989	9.204 (10.772)	0.85	0.441
X volume	5.32 (2.541)	2.09	0.075	3.011 (3.653)	0.82	0.456
Body mass	− 0.285 (0.09)	− 3.16	0.016*	− 0.142 (0.174)	− 0.81	0.461
Constant	1.986 (2.328)	0.85	0.422	1.427 (4.06)	0.35	0.743

* Significant effect (*p* < 0.05)

As can be observed only one of the 6 regression models yielded a significant outcome. It was the regression on PC3, a factor that was essentially extracted to explain the number of syllables per second and the number of syllable types per second, i.e., two dependent variables related to the rate of song production. In addition, the model only showed a significant relationship with body mass. So as a whole, syllable repertoire did not seem to be related in any close manner to the vocal control nuclei volumes.

## Discussion

Here, we provide a detailed quantitative characterization of song structure and organization for male and female canaries treated with sex steroids. These analyses identified dramatic sex differences in song and syllable-type diversity, and in syllable-type sequences (large and very large effect sizes, see Table 1). Overall, steroid-treated males sang songs that contain a higher number of syllable types when compared to similarly treated females. In addition, male songs follow more fixed patterns of syllable-type transitions than female songs. Taken together, these results agree with our previous reports showing that different measures of singing behavior and song structure (in the frequency and time domains) [22], and trill production [25] are activated more efficiently both by T and T plus E2 treatments in male than in female canaries. The present data also demonstrate that supplementing T with exogenous E2 does not enhance the responses of females to T as far as the syllable diversity and use is concerned. The groups additionally treated with E2 had been added because published papers indicated that in birds and mammals aromatase activity is lower in the brain of females compared to males even when treated with exogenous T [44, 45] and inhibition of aromatase activity affect singing motivation and song quality in canaries [32, 33, 46]. The lower aromatase in females might thus have explained why T is behaviorally less effective in females. The fact that no additional effect of E2 was observed here and in our previous paper [22] clearly argues against this interpretation and the differential reaction of males and females to a same treatment with T must therefore have another explanation (see “Ontogeny” section).

### Male songs are more diverse at multiple levels

Steroid-treated males and females concatenated syllable types into songs in similar ways to produce songs that superficially look similar. In particular songs produced under the influence of T have a similar duration in both sexes and they have a distribution of energy extending over the same bandwidth [22]. However male songs contained a higher number of different syllable types than female songs (on average 15.1 in males and 5.7 in females; Fig. 2C). The present analyses thoroughly captured song and syllable diversity at multiple organizational levels. First, at a global level, males exhibited larger song type (Fig. 2B) and syllable type (Fig. 2A) repertoires than females. There were also significant sex differences in metrics that captured within songs variability, such as the *number of syllable types per song* (Fig. 2C), *number of syllable types per second* (Fig. 2E), *and syllable versatility index* (Fig. 2G). Finally, we detected significant sex differences in *Levenshtein distance,* a variable that captures variation between successive songs in the sequence of specific syllable types they contain [38, 40]. Overall, females thus produced songs that are very stereotyped with many repeats of the same syllable type. It is important to note that most measures were affected by a substantial degree of individual variability and some females showed a greater diversity in their singing behavior, at times producing a number of song and syllable types closer to typical male songs [47]. Vallet and collaborators also pointed out these individual differences among testosterone treated females, observing that their repertoire size shows large individual differences.

Interestingly, steroid-treated males and females do not differ in syllable production as measured by the number of *syllables per song* (Fig. 2D) and number of *syllables per sec* (Fig. 2F). This is in line with a previous report showing no sex differences in the number of syllables per song and time vocalizing within songs (Fig. 2 in [22]), although we showed that males produce more songs and trills overall. Hence, sex differences seem to be present at the global level of song production, but also in a more subtle manner within songs where they are restricted to the diversity and organization and syllable use.

### Male songs show higher stereotypy in syllable-type transitions

We also found sex differences in patterns of transitions between syllable types. The network analysis of syllable sequences show that male songs follow more linear patterns of syllable-type transitions than female songs. We detected very significant sex differences in the two network variables used to characterize syllable-type transitions (large effect sizes with partial eta square equal to 0.477 and 0.499 for respectively network path length and network density; see Table 1). Male songs were associated with significantly longer paths than female songs (Fig. 3C). Thus, in male songs more transitions are required to connect any given pair of syllable types, which relates obviously to the fact that the syllable repertoire is larger and there are consequently more possible transitions (Syllable repertoire is indeed highly correlated with path length: *r* = 0.80 in males and *r* = 0.91 in females). In contrast, females showed significant higher values of *network density* (Fig. 3D). Thus female songs use a higher percentage of the possible transitions between syllable types in a given network. Their song is therefore less reproducible from one instance to the next even given the smaller size of their repertoire. In other words, in females there is more variability in syllable sequencing. Together, these results are reflecting song sequences that are less linear and more flexible in the transitions among syllable types in females as compared to males.

### Relationship with vocal control nuclei volumes

Very soon after the discovery of the vocal control system, Nottebohm and collaborators published a study identifying a significant positive correlation between repertoire size in 26 male canaries recorded at the peak of singing season and the volume of HVC or RA measured in brains collected 3 to 4 months later [48]. Some studies however failed to reproduce this result [49] and other experiments actually suggested that HVC volume was rather related to the singing motivation and the number of songs produced [50]. Accordingly there is a substantial amount of literature indicating that neurogenesis in HVC is controlled at least in part by the singing activity, in addition of course to the direct control by testosterone [51–55].

The present study failed to identify a significant relationship between syllable repertoire and volume of the three telencephalic vocal control nuclei in males as well as in females. These data therefore bring no support to the initial idea of Nottebohm and collaborators. Note however that all birds here were treated with exogenous steroids that could have artificially boosted the volume of the nuclei to its maximal level. The lack of correlation observed here should thus be considered with caution.

### Ontogeny

One can wonder how these stable sex differences affecting in a very specific manner some aspects of steroid-induced singing behavior, but not others, develop during ontogeny. It is well established that some aspects of the reproductive behavior of both birds and mammals are irreversibly differentiated following exposure to sex steroids during an early stage of development. In Japanese quail, exposure of females to their ovarian estrogens before day 12 of incubation irreversibly demasculinizes copulatory behavior. This effect can be mimicked in males by an injection of exogenous estradiol benzoate in the egg and blocked in females by an injection of an aromatase inhibitor that will prevent endogenous estrogen production [7]. The same mechanism appears to control the differentiation of copulatory behaviour in zebra finches [56] but the role of steroids in the control of the differentiation of the song system and of singing behaviour in this species remains unclear at present [7]. It is likely that sex steroids are implicated in this sexual differentiation [57, 58] but how exactly is unknown (for review see [7]).

Based on studies of a gynandromorphic zebra finch (half male-half female), it has also been demonstrated that genes affect sex differences of the song system independent of the differentiating effects of gonadal steroids [59]. Several genes that might be implicated in these “direct” effect have even been suggested see [7] for references) but the relative contribution of sex steroids and of “direct” genetic effects is not yet fully understood.

How these data apply to other songbird species, such as canaries, that show a less extreme sexual differentiation [60] is also not known but based on the repeated demonstration that some aspects of song cannot be fully masculinized by adult treatments with sex steroids, it appears very likely that these aspects are sexually differentiated in the organizational sense by the early action of sex steroids or genes. T-treated female canaries still possess smaller vocal control nuclei (HVC, RA and Area X; [21, 22]) than males and the two sexes exhibit major sex differences in gene expression in HVC [61] and RA [62]. Transcriptomes are also very different between spontaneously singing females and females singing after T treatment [61]. These studies [61] thus indicate that singing behavior correlates and is possibly caused, in part at least, by the expression of different genes in male and female canaries. Some of the male-biased genes are located on the Z chromosome, consistent with previous work demonstrating that dosage compensation is limited in birds (males have 2 Z chromosome while ZW females only have one [63–65]. However, the majority of sex-biased genes are located on autosomes. Their differential expression is thus presumably resulting from a secondary regulation by transcription factors or induced via epigenetic mechanisms by different life experiences that might include a differential early exposure to sex steroids as well as different interactions with their conspecifics. This idea should be experimentally tested via hormonal or genetic manipulation of young nestlings.

Finally, it is likely that, during ontogeny, females do not learn the same amount as males about conspecific song. Song learning is known to occur in female songbirds to permit male song recognition [66, 67] but females obviously do not practice these learned vocalizations as much as males do during the period of sensorimotor learning (plastic song). Therefore they probably do not consolidate this memory to the same extent as males. In addition, it has been demonstrated that in zebra finches, the interaction of the young nestling with its tutor plays a critical role in song learning [68]. Male and female nestlings can thus live in the same environment but be exposed to a different *Umwelt* [69]. This differential earning probably contributes to explain the more limited syllable repertoire of T-treated females as compared to males so that under the influence of T these females end up producing songs of the same length as males but they are unable to populate them with a variety of diverse syllables.

Acquisition of song in female and as a consequence in male canaries thus appears to be a very sophisticated process that is presumably based on genetic and endocrine mechanisms but also on specific learning processes during ontogeny. As such this represents an outstanding model to analyze the interaction between nature and nurture in the acquisition of a sophisticated learned behavior.

### Perspectives and significance

This study demonstrates that, even if female canaries can be induced to sing at male rates by a treatment with exogenous testosterone, some aspects of the song they produce, in particular the syllable repertoire and syllables organization into phrases, remain significantly different. The song of canaries is thus composed of aspects that are different between males and females not only because the adults are exposed to different concentrations of hormones. The song also contains aspects that cannot be masculinized by adult hormones and are thus probably sexually differentiated in the organizational sense. Future work should be devoted to the analysis of these organized aspects, in a first step by manipulating the neonate and juvenile endocrine environment, possibly in association with the exposure to an enriched acoustic environment.

## Supplementary Information

**Additional file 1: Table S1.** DataSet EBdS et al. Individual values of all results.

## Data Availability

The dataset(s) supporting the conclusions of this article is(are) included within the article and one additional Excel file located at the end of this manuscript (Table S1 Dataset EBdS).
